# Lentiviral gene therapy rescues p47^phox^ chronic granulomatous disease and the ability to fight *Salmonella* infection in mice

**DOI:** 10.1038/s41434-020-0164-6

**Published:** 2020-06-12

**Authors:** Andrea Schejtman, Walmir Cutrim Aragão-Filho, Simon Clare, Marta Zinicola, Maren Weisser, Siobhan O. Burns, Claire Booth, Hubert B. Gaspar, David C. Thomas, Antonio Condino-Neto, Adrian J. Thrasher, Giorgia Santilli

**Affiliations:** 1 Molecular and Cellular Immunology Unit, UCL Great Ormond Street Institute of Child Health, University College London, London, UK; 2 Department of Immunology, Institute of Biomedical Sciences, University of São Paulo, São Paulo, Brazil; 3 Wellcome Trust Sanger Institute, Wellcome Trust genome Campus, Hinxton, Cambridge UK; 4 Department of Immunology, Royal Free London NHS Foundation Trust, London, UK; 5 Institute for Immunity and Transplantation, University College London, London, UK; 6 Department of Paediatric Immunology, Great Ormond Street Hospital, London, UK; 7 Orchard Therapeutics, London, UK; 8 Immunity and Inflammation, Imperial College, London, UK

**Keywords:** Immunological disorders, Haematopoietic stem cells, Genetic transduction

## Abstract

Chronic granulomatous disease (CGD) is an inherited primary immunodeficiency disorder characterised by recurrent and often life-threatening infections and hyperinflammation. It is caused by defects of the phagocytic NADPH oxidase, a multicomponent enzyme system responsible for effective pathogen killing. A phase I/II clinical trial of lentiviral gene therapy is underway for the most common form of CGD, X-linked, caused by mutations in the gp91^phox^ subunit of the NADPH oxidase. We propose to use a similar strategy to tackle p47^phox^-deficient CGD, caused by mutations in *NCF1*, which encodes the p47^phox^ cytosolic component of the enzymatic complex. We generated a pCCLCHIM-p47^phox^ lentiviral vector, containing the chimeric *Cathepsin G*/*FES* myeloid promoter and a codon-optimised version of the human *NCF1* cDNA. Here we show that transduction with the pCCLCHIM-p47^phox^ vector efficiently restores p47^phox^ expression and biochemical NADPH oxidase function in p47^phox^-deficient human and murine cells. We also tested the ability of our gene therapy approach to control infection by challenging p47^phox^-null mice with *Salmonella* Typhimurium, a leading cause of sepsis in CGD patients, and found that mice reconstituted with lentivirus-transduced hematopoietic stem cells had a reduced bacterial load compared with untreated mice. Overall, our results potentially support the clinical development of a gene therapy approach using the pCCLCHIM-p47^phox^ vector.

## Introduction

Chronic granulomatous disease (CGD) is an inherited disorder of blood phagocytic cells that renders patients susceptible to certain bacterial and fungal infections and prone to sterile inflammatory complications [1]. The disorder is caused by mutations in genes encoding the Eros chaperone [2, 3] or any of the five subunits (gp91^phox^, p22^phox^, p40^phox^, p47^phox^ and p67^phox^) of the NADPH oxidase enzyme that mediates pathogen clearance via production of reactive oxygen species, activation of granule proteases and formation of neutrophil extracellular traps [4, 5]. Gp91^phox^ deficiency, which is responsible for X-linked CGD, accounts for the majority and generally most severe cases of CGD (65–70%) while p47^phox^ deficiency is the most common among autosomal recessive forms.

Despite enormous progress in diagnostic tests for CGD leading to more effective prophylactic treatments, the only curative option remains hematopoietic stem-cell transplantation (HSCT), although this is complicated by the risks of graft versus host disease and chemotherapy-related toxicity, especially in patients with underlying organ dysfunction/inflammation [6, 7]. HSC gene therapy for CGD was developed in the early 1990s with disappointing results, mainly as a result of gamma retrovirus-mediated insertional mutagenesis and loss of functional correction over time partly due to promoter methylation leading to transgene silencing [8–10]. Recently, a lentiviral gene-therapy trial for the X-linked form of CGD has shown stable reconstitution of NADPH oxidase activity in six out of eight patients with >1-year follow up [11]. The lentiviral vector used in the trial contains a chimeric promoter (CHIM) consisting of the 5′ minimal flanking region of the myeloid human genes *CTSG* (Cathepsin G) and *FES* (c-Fes) and preferentially drives high transgene expression in mature granulocytes and monocytes, the affected cells in CGD [12]. In this study, we propose to use a parallel lentiviral vector for the treatment of p47^phox^-deficient CGD (p47^phox^ CGD). p47^phox^ CGD accounts for ~25% of all CGD cases in western countries although its frequency rises in regions with high degree of consanguinity [13–15]. The disease is caused by mutations in the *NCF1* gene [16] (a deletion of GT at the start of the exon 2 accounts for almost 84% of cases [17]) encoding p47^phox^, the cytoplasmic component that, upon phosphorylation and translocation to the membrane, coordinates the assembly of the cytosolic subunits of NADPH oxidase resulting in the activation of the enzymatic complex. p47^phox^ CGD has been historically associated with a milder phenotype compared with X-CGD as the majority of patients have residual superoxide production; however, infections are still a major cause of mortality and morbidity and patients still suffer from gut disease and inflammation [18].

In this study, we tested the pCCLCHIM-p47^phox^ lentiviral vector for its ability to restore p47^phox^ expression and NADPH oxidase function in human p47^phox^-deficient cells and in a murine model of the disease. To evaluate the ability of the newly developed gene therapy protocol to confer protection against pathogens in vivo, p47^phox^-null mice  reconstituted with lentivirus-transduced cells were challenged with *Salmonella* Typhimurium (one of the major threats for CGD patients in developing countries and the leading cause of septicaemia in a European cohort [19]) and showed that the infection burden is significantly reduced, although not completely eradicated, after gene therapy. Taken together, our results demonstrate that lentiviral gene therapy may be a valuable alternative to HSCT for patients with p47^phox^ CGD lacking a suitable donor.

## Materials and methods

### Vector construction

A codon-optimised form of the *NCF1* coding sequence (GeneArt, Regensburg, Germany) was initially cloned into the pCR™ Blunt II-TOPO^®^ vector (ThermoFischer Scientific) and subsequently removed by MluI (*blunt*)-XhoI restriction sites and cloned into the BamHI (*blunt*)-SalI site of the pCCLCHIM-GFP vector [12] in place of GFP. An HpaI-XhoI fragment containing the CHIM-p47^phox^ cassette was then sub-cloned in place of the CHIM-gp91^phox^ in the clinical G1XCGD vector [20] to form the pCCLCHIMcop47_WPRE4 (hereby referred as pCCLCHIM-p47^phox^). A truncated version of the vector pCCLCHIM-p47^phox^ lacking the first 600 bp of the codon-optimised *NCF1* coding sequence was used for mock transduction. The sequence of the transcriptional cassette for the full length and truncated version of the pCCLCHIM-p47^phox^ is shown in Supplementary Fig. 1.

### Cell culture and differentiation

Human PLB985, an acute myeloid leukaemia cell line (DSMZ GmbH), was cultured in RPMI 1640 medium (ThermoFisher Scientific), 10% FBS (ThermoFisher Scientific) and 1% Penicillin-Streptomycin (ThermoFisher Scientific) at a concentration of 300,000 cells/ml. For granulocytic differentiation, PLB985 (250,000 cells/ml) were seeded in RPMI 1640 medium containing 0.25% FBS, 0.5% di-methyl formamide (Sigma-Aldrich, St. Louis, Missouri) and 1x Nutridoma-CS (Roche, Basel). Media was added every other day for 4 days in order to maintain the cell concentration at 250,000 cells/ml.

Lineage negative cells were isolated from mouse femur, tibia and hips using the Lineage Cell Depletion Kit (Miltenyi Biotec, Auburn, CA). Cells were cultured in Stem Spam media (StemCell Technologies, Vancouver, Canada) enriched with 100 ng/ml mFlt-3, 100 ng/ml mSCF and 25 ng/ml hTPO (all cytokines were from Peprotech, Rocky Hill, NJ) during transduction. For granulocytic differentiation, cells were cultured at a concentration of 300,000/ml for 9 days in RPMI, 20% FBS supplemented with 100 ng/ml of granulocytic colony-stimulating factor (Peprotech).

Monocyte-derived macrophages (MDMs) were obtained as previously described in Chiriaco et al. [21]. Briefly, monocytes were isolated from peripheral blood mononuclear cells of p47^phox^-deficient CGD patients through positive selection using CD14+ microbeads and MS columns (Miltenyi Biotec) following the manufacturer’s instructions. Patients signed a written informed consent in accordance with the Declaration of Helsinki and ethical approval NRES committee London (REC reference 04/Q0501/119). Cells were cultured for 7 days in RPMI 1640 containing 10% FBS, 1% P/S and 50 ng/ml of macrophage colony-stimulating factor (Peprotech).

### Vector production

Lentiviral vector supernatants were produced by transiently co-transfecting 293T cells with the polyethylenamine (PEI, Sigma-Aldrich) and the DNA complexes containing the transfer vector, the vesicular stomatitis virus-G envelope plasmid (pMD.G2) and packaging plasmid (pCMVdR8.74). The viral supernatants were concentrated by ultracentrifugation at 24,000 rpm for 2 h at 4 °C. The viral titre was determined by transducing PLB985 cells with serial dilutions of the vector preparation and analysing vector copy number (vcn) by quantitative PCR, 3 days post transduction.

### Lentiviral transduction

For lentiviral transduction of MDMs, virion protein X incorporated into virus-like particles (Vpx-VLP) were produced by co-transfecting 293T cells with pMD.G2, the VSV-G containing plasmid and the Simian immunodeficiency virus-derived packaging plasmid SIV3+ (a kind gift of Dr Cimarelli) [22]. MDMs (7 days into the differentiation protocol) were transduced for 24 h with the pCCLCHIM-p47^phox^ vector at multiplicity of infection (MOI) 5 or 50 after 6-h incubation with Vpx-VLP particles. Three days post transduction cells were incubated for 10 min in Trypsin-EDTA (ThermoFisher Scientific) and gently scraped off the culture plate for FACS analysis by LSRII (Becton Dickinson). Lentiviral transduction of PLB985 cells or murine lineage negative cells was performed by adding the vector supernatant in culture media for 24 h.

### p47^phox^ detection and flow cytometry analysis

MDMs were stained with anti-human CD11b-APC (clone M1/70, Biolegend), washed in PBS/0.5% BSA, fixed and permeabilized using the Perm/Fix kit (Nordic MUbio) according to manufacturer’s instructions. Cells were incubated with the anti-human p47^phox^ antibody (clone 1, Becton Dickinson) in permeabilization buffer for 20 min followed by staining with a secondary FITC-conjugated mouse IgG (Beckman Coulter) before analysis in the LSRII instrument (BD Bioscience).

Murine cells (1 × 10^6^ cells) were initially co-stained with a mix of anti Gr-1-APC (clone RB6-8C5, Biolegend) and anti CD11b-PercpCy5.5 (clone M1/70, Bioscience), or with a mix of anti B220-APC (clone RA3-6B2, Bioscience) and anti-CD3-PeCy5 (clone 17A2, Biolegend) in the presence of γFc blocking and then intracellularly stained for p47^phox^ as described above. PLB985 clones were analysed for p47^phox^ expression after granulocytic differentiation by staining with an anti-human CD11b-PE (clone M1/70, Biolegend) followed by intracellular staining with anti-human p47^phox^-(clone 1) APC labelled by Beckton Dickinson services. FACS analysis was performed in CyAn^TM^ ADP (Beckman Coulter).

### Dihydrorhodamine test (DHR)

Differentiated PLB985 cells (5 × 10^5^) were stained with the myeloid marker CD11b-APC (clone M1/70, Biolegend). Cells were then incubated with 2.9 μM of DHR 123 (DHR; Sigma-Aldrich) in 500 μl of PBSgg (0.05% gelatin, 0.09% D-glucose) containing 150 U/ml of catalase (Sigma-Aldrich) for 15 min at 37 ^o^C and subsequently activated with 1 μg/ml of PMA (Sigma-Aldrich) for further 15 min at 37 ^o^C. Cells were kept on ice and analysed in CyAn^TM^ ADP (Beckman Coulter) within 30 min.

For murine cells, 50 μl of blood or 1 × 10^6^ bone marrow cells were stained with APC labelled anti-murine CD11b (clone M1/70, Biolegend) and anti-murine Gr-1 (clone RB6-8C5, Biolegend) antibodies before being subjected to a DHR test as above described. DHR positivity was calculated by gating on the cells with high SSC and high expression of CD11b/Gr-1 defined as granulocytes.

### Primary and secondary transplantation of p47^phox^ null mice

p47^phox^ null mice (B6 [Cg]-Ncf1^m1J/J^) were kindly provided by Dr Ulrich Siler, University of Zurich and bred at the University College London-Bloomsbury Campus. C57BL/6 mice were purchased from Charles River Laboratories, Netherlands. All procedures were performed following the Animals Scientific Procedures Act 1986, Amendment Regulations 2012 (ASPA). Sample size was determined based on previous experience.

Lineage negative cells were isolated from 6 to 8 weeks old male and female mice and cultured for 24 h in the presence of the truncated pCCLChim-Δ600p47^phox^ vector (Mock) or the pCCLChim-p47^phox^ vector, MOI 100. Cells (6.5 × 10^5^) were intravenously injected into lethally irradiated (9.5 Gy: split dose of 6 and 3.5 Gy into two consecutive days) recipient mice, 4–5 h after the last irradiation. Transplanted mice were monthly bled to monitor reconstitution levels. Four to six months post-transplantation (depending on the experiment) mice were sacrificed and peripheral blood, bone marrow, spleen and thymus were harvested for analysis. For secondary transplantation, we harvested ~4 × 10^7^ cells from hips, femurs and tibia of six gene-therapy-treated animals and injected half in the tail vein of newly prepared recipient mice (1:2 ratio).

### Salmonella challenge

For the *Salmonella* challenge 5 × 10^5^ colony-forming unit (CFU) of *Salmonella enterica serovar* Typhimurium M525 in sterile PBS (Sigma-Aldrich) was i.v.-administered to the mice. Animals were monitored daily for 7 days and sacrificed if they lost ≥20% of weight, according to the NC3Rs recommendation. In a different set of experiments animals were left for 3 days after the *S*. Typhimurium injection and sacrificed at day 3 post infection. Quantification of bacterial counts in peripheral blood, spleen and liver were performed by serial dilution and plating onto agar plates (ThermoFisher Scientific Oxoid).

### Statistical analysis

For comparison among two groups we used the Student’s *t* test. For comparisons among more than two groups we used the analysis of variance (ANOVA) followed by Sidak or Bonferroni post-test correction. The Kaplan–Meier survival curve was analysed using the log-rank (Mantel–Cox) test. *p* values < 0.05 were considered statistically significant. All tests were performed using GraphPad Prism version 7.

## Results and discussion

### Lentiviral gene therapy restores p47^phox^ expression in human cells

**Fig. 1:**
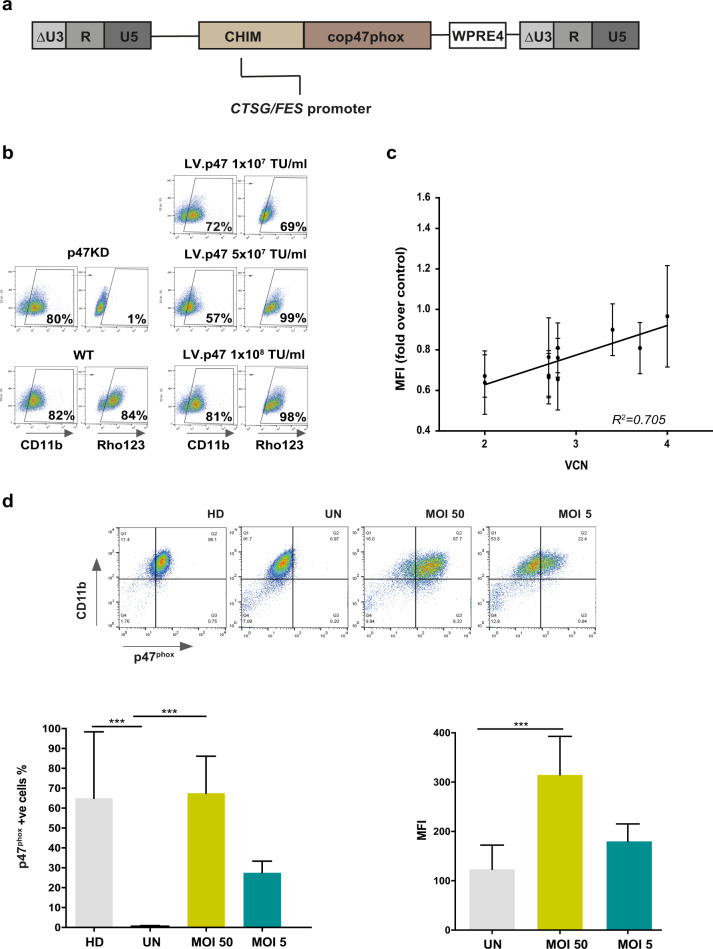
Gene therapy rescues the p47^phox^ CGD phenotype in human cells. **a** Schematic representation of the provirus showing the Chimeric promoter (CHIM), the codon-optimised *NCF1* coding sequence (cop47phox) and the WPRE4. **b** Dihydrorhodamine test (Rho123) in the p47^phox^ knock-down PLB985 cells (p47KD) that were transduced with the pCCLCHIM-p47^phox^ lentiviral vector  (LV.p47) at different multiplicity of infections (MOIs) and differentiated into granulocytes (CD11b+) with di-methyl formamide. Representative FACS plots out of three experiments. **c** Clonal populations (*n* = 11) were obtained by single cell sorting PLB985 p47KD cells 1 week after transduction with 1 × 10^7^ transducing units (TU)/ml of the pCCLCHIM-p47^phox^ vector. The plot shows on the *Y*-axis levels of p47^phox^ expression, as measured by the mean fluorescence intensity (MFI) over wild-type cells and on the *X*-axis vector copy number for each clone (*n* = 3 experiments; data are presented as mean ± SD; *R*^2^ = squared Pearson’s coefficient of correlation (*p* = 0.0012)). **d** Representative FACS plots showing p47^phox^ expression after lentiviral transduction with the pCCLChim-p47^phox^ vector (MOI 5 and 50) in monocyte-derived macrophages (MDMs) from one p47^phox^ CGD patient (upper panel). p47^phox^ expression in untransduced cells from the same patient (UN) and from a healthy donor (HD) are shown as negative and positive control, respectively. Lower left panel: percentage of p47^phox^ positive cells among the CD11b^+ve^ population. Lower right panel: p47^phox^ expression levels as measured by MFI. Data are presented as mean ± SD (*n* = 6; ***p < 0.001, one-way ANOVA followed by Bonferroni post-test correction).

We generated a self-inactivating lentiviral vector containing the ‘chimeric’ myeloid promoter (CHIM) [12] and a codon-optimised version of the *NCF1* coding sequence (cop47phox). A schematic representation of the pCCLCHIM-p47^phox^ vector is shown in Fig. 1a.

In order to evaluate the efficacy of our lentiviral gene transfer in human cells, we created a myeloid cell line with a defective p47^phox^ protein by electroporating PLB985 cells with a Cas9: guide RNA ribonucleoprotein complex targeting exon 1 of the *NCF1* locus (Supplementary information and Supplementary Fig. 2a). The cell line, named p47KD, lacks NADPH oxidase activity (Supplementary Fig. 2b) and shows a dramatically reduced p47^phox^ protein expression upon myeloid differentiation when compared with wild-type cells (Supplementary Fig. 2c, d).

We firstly confirmed the ability of the pCCLCHIM-p47^phox^ lentiviral vector to restore NADPH oxidase function in the p47KD cells by a DHR flow cytometric assay measuring production of reactive oxygen species (Fig. 1b). We next derived clonal populations from cells transduced with the pCCLCHIM-p47^phox^ vector to correlate levels of p47^phox^ expression with the vcns of each clone (Fig. 1c). We found a positive correlation (Pearson’s *r* = 0.839; *R*^2^ = 0.705) between vcn/cell and expression of p47^phox^, suggesting that the levels of p47^phox^ found in each clone are not influenced by position effects. This is in line with previous reports [12, 23] showing stable and vector-dependent expression of a transgene driven by the chimeric promoter in the context of lentiviral vectors.

Due to the difficulty in recruiting hematopoietic stem and progenitor cells (HSPCs) donors among the small cohort of p47^phox^ CGD patients in UK, we tested the gene therapy protocol in macrophages derived from CD14+ monocytes (MDMs) that were isolated from peripheral blood of patients. We transduced MDMs using a low and high MOI and evaluated the percentage of p47^phox^ positive cells, as well as the levels of transgene expression (Fig. 1d). Transduction was performed in the presence of the Vpx, used to overcome the interference that the monocytic SAMHD1 protein poses on reverse transcription [24]. As expected, lentiviral transduction using MOI 5 resulted in a lower percentage of p47^phox^-positive macrophages compared with MOI 50 (Fig. 1d, upper and lower left panel), but the levels of expression were similar to those found in healthy donors (Fig. 1d, lower right panel) indicating good performance of the vector.

### Lentiviral gene therapy restores p47^phox^ expression and NADPH oxidase function in a mouse model of p47^phox^-deficient CGD

**Fig. 2:**
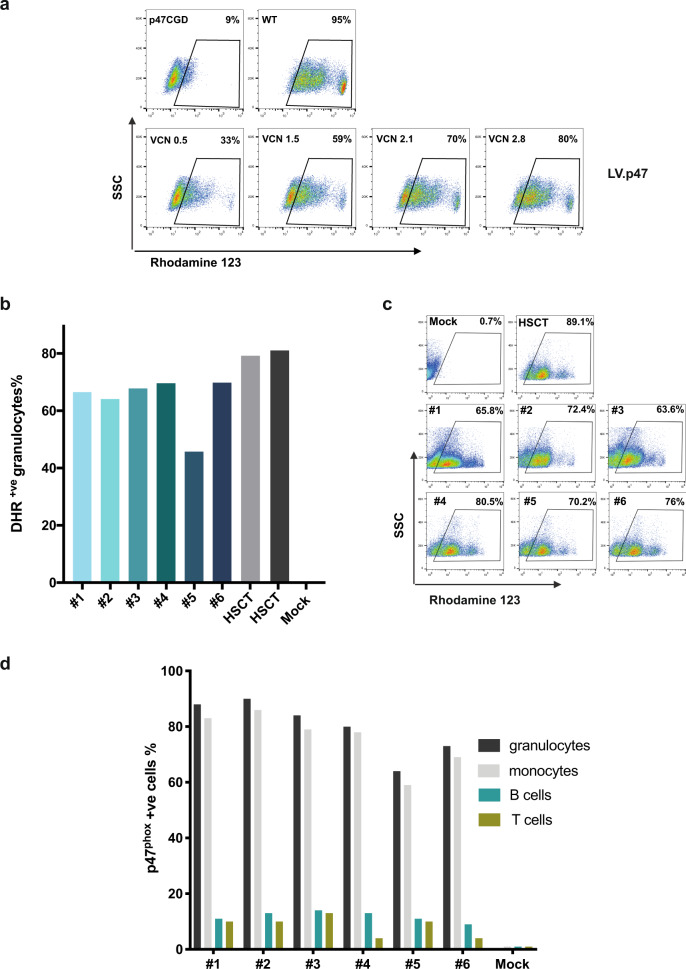
Gene therapy rescues  the  p47^phox^ CGD phenotype in a murine model of p47^phox^-deficient CGD. **a** Quantification of NADPH oxidase activity (as assessed by a DHR test; rhodamine 123) in lineage negative cells that have been transduced with different amounts of pCCLCHIM-p47^phox^ (LV.p47) and undergone granulocytic differentiation. Vector copy number/cell (vcn) is shown in each plot. Representative FACS plots out of two replicates. **b** DHR assay in peripheral blood granulocytes from gene-therapy-treated mice (#1-#6), 5 months post transplantation. DHR plots from p47^phox−/−^ mice transplanted with wild-type (HSCT) or mock-transduced p47^phox−/−^ (Mock) cells are also shown as positive and negative controls. **c** FACS plots showing a DHR assay in bone marrow granulocytes from gene-therapy-treated mice (#1-#6), 6 months post transplantation. **d** Percentage of p47^phox^ positive cells (as assessed by antibody immunostaining and FACS analysis) out of granulocytes (CD11b+/Gr-1^high^) and monocytes (CD11b+/Gr-1^low^) from the bone marrow, and T cells (CD3^+^) and B cells (B220^+^) from the spleen of transplanted animals (#1-#6), 6 months post gene therapy. A mock transplanted mouse is shown as negative control for the staining.

We next investigated the ability of lentiviral gene therapy to rescue the enzymatic activity of the NADPH oxidase in a mouse model of p47^phox^-deficient CGD. We transduced lineage negative HSPCs (Lin^−ve^) from p47^phox−/−^ mice with increasing amounts of the pCCLCHIM-p47^phox^ lentiviral vector as shown by increasing vector copies in transduced cells (Fig. 2a). Vector transduction did not alter the ability of p47^phox−/−^ Lin^−ve^ to form myeloid colonies in a CFU assay (Supplementary Fig. 3a). Upon myeloid differentiation, granulocytes containing ≥2 vector copies per cell exhibited DHR levels close to wild type suggesting that the strength of the chimeric promoter is comparable to that of the endogenous p47^phox^ promoter (Fig. 2a and Supplementary Fig. 3b).

In order to test our gene therapy protocol in vivo, we transduced p47^phox−/−^ Lin^−ve^ with the lentiviral vector at MOI 100 for 24 h (resulting in five vector copies per cell) and transplanted the cells into lethally irradiated p47^phox−/−^ recipients. As control, p47^phox−/−^ mice were also transplanted with mock-transduced p47^phox−/−^ (Mock) or with wild-type (HSCT) Lin^−ve^ cells. Analysis of peripheral blood samples 5 months post transplantation showed a percentage of DHR positive cells ranging from 46 to 70% in six out of eight animals reconstituted with lentivirus-transduced cells (Fig. 2b). We were not able to detect any functional neutrophils in the blood of two animals (data not shown) correlating with almost undetectable vcns in hematopoietic organs (Table 1, experiment A). Of note the same two animals did not have any DHR positive cells in their blood, 1 month after transplantation, suggesting that they were not engrafted with donor cells probably due to technical reasons (Supplementary Fig. 4a).

**Table 1:** Vector copy number in the peripheral blood and other hematopoietic organs of gene therapy mice (primary and secondary).

Experiment A	Mice	PB	BM	SP	Thy
Primary transplant	#1	6.35	6.3	6.97	7.62
	#2	5.54	6.15	5.54	5.06
	#3	2.55	6.03	6.22	7.93
	#4	5.94	3.96	4.79	3.49
	#5	4.72	6.62	5.53	5.34
	#6	6.12	6.57	5.73	6.18
	#7	0.20	0.00	0.10	0.02
	#8	0.00	0.40	0.00	0.00
Secondary transplant	#1	5.87	Underwent Salmonella challenge		
	#2	2.91			
	#3	3.99			
	#4	4.01			
	#5	5.04			
	#6	2.78			
	#7	1.55			
	#8	4.06			
	#9	4.98			
	#10	4.16			

Experiment B	Mice	PB	BM	SP	
Primary transplant	#1	1.31	1.57	1.1	
	#2	1.30	0.83	0.96	
	#3	3.66	Underwent Salmonella challenge		
	#4	4.68			
	#5	2.39			
	#6	1.47			
	#7	5.44			
	#8	5.20			
	#9	7.25			
	#10	5.95			
Secondary transplant	#1	0.35	0.15		
	#2	0.96	0.59		
	#3	0.53	0.29		
	#4	1.47	0.97		

Experimental mice were sacrificed 6 months post transplantation. Analysis of superoxide production in the bone marrow of transplanted mice confirmed that gene therapy rescued a high number of functional neutrophils in all engrafted mice (Fig. 2c). We then quantified the levels of p47^phox^ expression in different hematopoietic cell lineages (Fig. 2d). As expected, due to the nature of the chimeric promoter, a high percentage of neutrophils and monocytes expressed the p47^phox^ transgene in comparison with a lower percentage of B cells and T cells (Fig. 2d and Supplementary Fig. 4b), mirroring the physiological pattern of p47^phox^ expression.

**Fig. 3:**
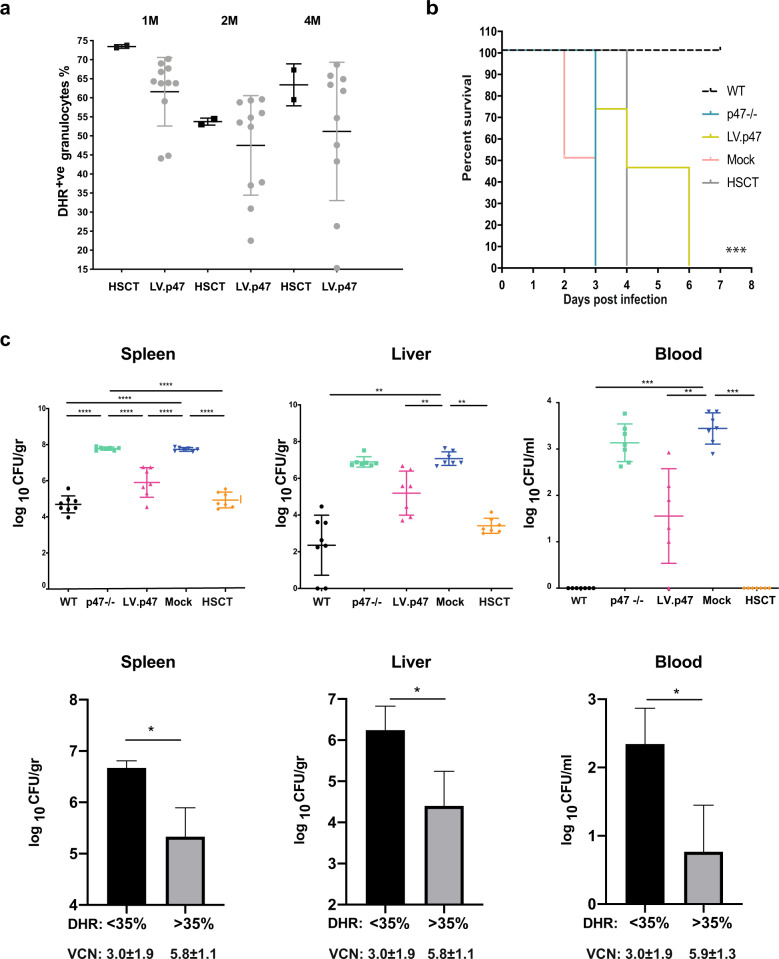
Survival and bacterial count in response to *Salmonella* Typhimurium infection. **a** Analysis of NADPH oxidase function (DHR test) in peripheral blood granulocytes from secondary-transplanted p47^phox−/−^ mice at 1, 2 and 4 months post transplantation. Shown are mice transplanted with bone marrow cells taken from wild-type (HSCT) or gene-therapy-treated (LV.p47) animals. One mouse in the LV.p47 group was sacrificed at 3 months due to the development of a B-cell leukaemia. Vector copy number in the peripheral blood of LV.p47 secondary-transplanted animals (4 months post transplantation) is shown in Table 1, experiment A. **b** Kaplan–Meier curve showing the survival of HSCT (*n* = 2), Mock (*n* = 2) and LV.p47 (*n* = 10) secondary recipients after intravenous injection of 5 × 10^5^ CFU of *Salmonella* Typhimurium M525. p47^phox−/−^ mice (*n* = 10) and C57BL/6 wild-type mice (*n* = 10) were used as control of the experiment. ***p < 0.001 denotes the significant difference between the LV.p47 and p47^phox−/−^ groups. Statistical analysis was made by the log-rank test. **c** p47^phox−/−^ mice were transplanted with lentivirus-transduced (LV.p47) or mock-transduced (Mock) p47^phox−/−^ and with wild-type (HSCT) Lin^−ve^ cells. Four months post-gene therapy mice were challenged with *S*. Typhimurium. Three days post-infection bacterial load was assessed in spleen, liver and blood. Normal C57BL/6 (WT) and p47^phox−/−^ mice were used as control for the experiment. Upper panels: bacterial counts (expressed as CFU/gr or CFU/ml) in the spleen (left), liver (middle), peripheral blood (right) of LV.p47 (*n* = 7), HSCT (*n* = 8) or Mock (*n* = 8), p47^phox−/−^ (*n* = 8) and WT (*n* = 8) mice. Data are presented as mean ± SD. Statistical analysis was performed with one-way ANOVA with Sidak’s post-test correction; **p < 0.001, ***p < 0.001, ****p < 0.0001. Lower panels: bar graphs showing the mean ± SD of bacterial counts found in the spleen (left), liver (middle) and blood (right) of mice with high DHR > 35% (*n* = 4 for spleen and liver; *n* = 3 for blood as one outlier was removed) and low <35% DHR (*n* = 3). DHR was determined in the blood of LV.p47 mice 4 months post transplantation. Statistical analysis was performed using a Student’s *t* test, *p < 0.05.

The longevity of our treatment was tested by transplanting bone marrow cells from the gene therapy, Mock and HSCT groups into secondary p47^phox−/−^ recipient mice and monitoring the percentage of functional neutrophils over time for 4 months (Fig. 3a). Vcns in primary- and secondary-transplanted animals are shown in Table 1 (experiment A). In contrast to other studies where p47^phox−/−^ mice reconstituted with gene-corrected cells have been monitored for only a few weeks post transplantation [25] we observed mice for almost 1 year (when taking into account primary and secondary mice) without witnessing any significant decrease in neutrophil function. Our study suggests that with our protocol we are able to transduce HSPCs without affecting their engraftment or repopulation ability, a reassuring finding given the difficulties experienced in the first human trials with gamma retroviral vectors for CGD [26] where correction was mainly transient, in the absence of clonal expansion.

### Lentiviral gene therapy improves the outcome of *Salmonella* Typhimurium infection in p47^phox^-deficient CGD mice

We completed the efficacy evaluation of the pCCLCHIM-p47^phox^ lentiviral vector by assessing whether gene-therapy could limit infection in mice challenged with *Salmonella.* We have chosen to use systemic administration of *Salmonella* Typhimurium mainly for its robust reliability, as demonstrated in the gp91^phox−/−^ and p22^phox−/−^ mouse models [27]. Secondary graft recipients alongside with C57BL/6 wild-type and p47^phox−/−^ mice were intravenously injected with 5 × 10^5^ CFU of attenuated *Salmonella* Typhimurium. Upon bacterial challenge, the gene therapy group (LV.p47) survived longer than the Mock or p47^phox−/−^ groups, similar to secondary recipients of wild-type cells (HSCT). Untreated C57BL/6 wild-type animals were resistant to the infection (Fig. 3b). The severity of this infection model in CGD mice has already been described [27] and confirmed in this study by the rapid death of p47^phox−/−^ mice.

The efficacy of gene therapy was also reflected in a reduction in bacterial load when additional gene-therapy-treated mice were sacrificed 3 days post *Salmonella* Typhimurium infection. CFU counts in spleen, liver and peripheral blood of p47^phox−/−^ mice reconstituted with lentivirus-transduced cells were significantly lower than those measured in p47^phox−/−^ or mock-treated mice although higher than those found in C57BL/6 mice and in p47^phox−/−^ mice transplanted with wild-type cells (Fig. 3c upper panels). The effect was more pronounced in mice with >35% of DHR positive granulocytes in their peripheral blood (Fig. 3c; lower panels). This result is in keeping with a previous report showing that transplanted X-linked CGD mice with 30% donor chimerism have a significantly reduced bacterial load, upon *Salmonella* infection, in comparison with gp91^phox^-null animals [28] and suggests that the levels of NADPH-oxidase activity that we obtain in lentivirus-transduced phagocytic cells is sufficient for clinical effect. Of note, a recent study conducted on 162 carriers of X-linked CGD reports that 20% of functional neutrophils are required to confer protection against life-threatening infections [29], although lower levels also have very significant effects [30]. Secondary transplantation experiments confirmed the longevity of the gene therapy treatment (Supplementary Fig. 4c). Vcns in the blood of gene-therapy-treated mice shown in Fig. 3c and Supplementary Fig. 4c are presented in Table 1 (experiment B).

Taken together, the results presented here show that our lentiviral gene therapy protocol by the use of the pCCLCHIM-p47^phox^ vector can stably restore p47^phox^ expression and NADPH-oxidase function in relevant cells and ameliorate infections.

Targeted insertion of the therapeutic *NCF1* coding sequence in the *AAVS1* safe harbour locus [31] or targeted correction of the GT base pair deletion at the start of *NCF1* exon 2 [32] have recently been proposed as alternatives to a gene addition protocol. However, despite rapid advances in the gene editing field for monogenic blood disorders, protocols that contemplate the need for cell proliferation/activation in order to promote homology-directed repair could prove unsuited for HSC that are already compromised by chronic inflammation and may show an accelerated loss of stem-cell phenotype upon culture [33]. We believe that a lentiviral transduction protocol that entails short-term ex vivo cell culture will offer a rationale treatment option for p47^phox^ CGD.

## Supplementary information

Supplementary Information

Supplementary Figure 1

Supplementary Figure 2

Supplementary Figure 3

Supplementary Figure 4

Supplementary Figure Legends
