# Supplementary material for: Lentiviral gene therapy rescues p47phox chronic granulomatous disease and the ability to fight Salmonella infection in mice
Source: Gene Ther. 2020 Jun 12;27(9):459–69. doi: 10.1038/s41434-020-0164-6 (PMC7500983; doi:10.1038/s41434-020-0164-6)
Supplement: Supplementary file 1 — Supplementary Information [file 41434_2020_164_MOESM1_ESM.docx]

**Supplementary Information**

**Supplementary Materials & Methods**

**Generation of p47^phox^ defective PLB985 (p47KD)**

To obtain the p47^phox^ defective cell line, PLB985 cells were electroporated using the Neon Transfection system (Thermo Fisher Scientific; program KG-1) with a Cas9 (Invitrogen, Carlsbad, CA) ribonucleoprotein complex targeting *NCF1* exon 1 (target sequence + PAM is highlighted in Supplementary Figure 2A). The gRNA was synthesized using the GeneArt Precision gRNA Synthesis Kit according to manufacturer’s instructions (Thermo Fisher, Carlsbad, CA). After one week in culture, the bulk population was single cell sorted (FACS Aria III FCF; Beckton Dickinson) in 96 well plates for clonal expansion. After 20 days, a mirror 96 well plate was created and induced to undergo myeloid differentiation before assessing the ability of the clones to produce superoxide with a Nitroblue tetrazolium test (NBT).

**Nitroblue tetrazolium test (NBT)**

Differentiated PLB985 clones were loaded with a solution containing 1 µg/mL of Nitroblue tetrazolium salt (Sigma-Aldrich) and 1 µg/mL of PMA (Sigma-Aldrich). Cells were incubated at 37 ^o^C for 20 min before scoring the ones containing or not deposits of blue formazan under light microscopy.

**Western blot analysis**

For the detection of p47^phox^ by Western blot, PLB985 wild type, X-CGD (as further control) and p47KD were induced to undergo myeloid differentiation as previously described. 25 µg of total proteins were separated using NuPAGE 4-12% Bis-Tris gel (Thermo Fisher Scientific) and transferred onto PVDF membrane. The membrane was immunoblotted with a mouse anti human p47^phox^ antibody (BD Bioscience) followed by an HRP conjugated rabbit anti-mouse IgG antibody (Thermo Fisher Scientific). The expected 47 kDa band on the membrane was visualised using the SuperSignal^TM^ West Pico Plus Chemiluminiscent substrate (Thermo Fisher Scientific) with the GeneGnome chemiluminescence imaging system (SynGene).

**Sanger sequencing evaluation of p47KD cells**

The *NCF1* locus has two pseudogenes (*NCF1B*, *NCF1C*), each containing a deletion of the GT base pair (ΔGT) at the start of exon 2, that could equally be targeted by the Cas9 ribonucleoprotein complex chosen for this study. Therefore, we decided to use a retrotranscribed cDNA to obtain PCR products that encompassed the whole coding sequence of *NCF1* to analyse INDELs specifically in the *NCF1* locus*.* RNA from the p47KD clone was extracted using the RNeasy Mini Kit (Qiagen) and then retrotranscribed in cDNA using SuperScript^®^ III First-Strand Synthesis System for RT-PCR according to manufacturer instructions (Thermo Fisher Scientific). A PCR reaction was performed using the following primers: forward- 5’_CCAGTGCATTTAAGGCGCAGC_3’ and reverse- 5’_GCTCTCGCTGCACCGGTTCAG_3’ and the amplified fragment was then sub-cloned into pCR™ Blunt II-TOPO^®^ vector (Thermo Fischer Scientific). Minipreps from 23 colonies were sent for Sanger sequencing.

**Vector copy number (VCN) assessment**

To determine VCN, genomic DNA was extracted using the QIAmp DNA Blood kit (QIAGEN, Germany) following the manufacturer’s instructions and a real-time PCR (qPCR) was performed using Absolute qPCR ROX mix (Thermo Fisher, Carlsbad, CA). For detection of VCN in human cells we used the following primers/probes for HIV: HIV psi forward 5’_CAGGACTCGGCTTGCTGAAG_3’ and reverse 5’_TCCCCCGCTTAATACTGACG_

3’; the HIV psi probe sequence was 5’_FAM_CGCACGGCAGGCGAGG_TAMRA_3’; and the following primers/probe for human albumin: Albumin forward 5’_GCTGCTATCTCTTGTGGGCTGT_3’ and reverse 5’_ACTCATGGGAGCTGCTGGTT

C_3’; the Albumin probe sequence was 5’_VIC_CCTGTCATGCCCACACAAATCTCTCC

_TAMRA_3’. For the detection of VCN in murine samples we used the following primers/probes for WPRE: forward 5’_TGTATCATGCTATTGCTTCCCG_3’ and reverse 5’_GTTGCGTCAGCAAACACG_3’; the WPRE probe sequence was 5’_FAM_TTATGAGGAGTTGTGGCCCGTTGT_TAMRA_3’; and the following primers/probe for Titin: forward 5’_ACCGAGAGAGGTGGTATTGA_3’ and reverse 5’_AGGATGCCTCCTGCTTAGA_3’; the Titin probe sequence was 5’_HEX_AGCGTCTC

GTCTCAGTCAGTCCAA_ TAMRA _3’.
