# Supplementary material for: Lentiviral gene therapy rescues p47phox chronic granulomatous disease and the ability to fight Salmonella infection in mice
Source: Gene Ther. 2020 Jun 12;27(9):459–69. doi: 10.1038/s41434-020-0164-6 (PMC7500983; doi:10.1038/s41434-020-0164-6)
Supplement: Supplementary file 2 — Supplementary Figure 1 [file 41434_2020_164_MOESM2_ESM.pdf]

Supplementary Figure 1

Chimeric promoter
**CTGCCAGCTTTCTTGCTTTGCTGGAGTATTCTGGAATTTGATGGGTTGAGGGTTCTGGACACAATGCCCCAAGCCCCTTCCTTGTTGTGC
TGGGTTCCTATTTCTGCTCTCGGCACTGACTTAGCAGCTGCTCAAGAGCTCACCATGTTGGCTTGGATTACACGGTCTCACCCACATCTC
CGGCAGTTTGTGGGCAAACTTCCTGAGCAGCCTTGGGTGATGAAACCTTTCATGGTAGCAGGAGAATGGGACTGTGAATTCTCAATCCC
CTGTCCCCACCCCTTCCTTCCTCTCTCAGGGCCTTGCTGTCTAGGAGGAGGGAGCACAGCAGCAACTGACTGGGCAGCCTTTCAGGAA
AGGCTAGCCCGGGCTCGATCGAGAAGCTTGATAATTCCGTGAGGTGGGGAGGGCTGGGACCAGGGTTCCCTCTTTCTCTTCTGCGGTG
GCCCTGGCCTGGTGCTAGGACTGCGCGCCTCCCCTCAGTACCCGCGGACACCCTGGGCTTCCCTGGCCCAGCATCTGCCTGGGGCC
TCGCCCTGGGCTCCCCCTCCTGACCCCCACCTTGCGCCCCTTCCCGGTGTTCCCGGGGCGCTGCCGGGCCCTGGGGCCTGCGGGGC
GCGGGCGGCTCTTGGCTGGGCCATTCTTTCCCGGCCCCCTCCTCCCTTCCGTTTCCGTGGCCGTGCGGCCGGCTAGAGGCTGCGGCC
CAGCGCGGAGCAGGGGGGCTGGCAGGCGTCGGGGCGGTCGGGCCGGTCCCGCCCGCCCCTTCCCCTCCACAGGCCCGCCCCGGGG
CCTGGGCCAACTGAAACCGCGGGAGGAGGAAGCGCGGAATCAGGAACTGGCCGGGGTCCGCACCGGGCCTGAGTCGGTCCGAGGC
CGTCCCAGGAGCAGCTGCCC GAAGGGCGAATTGGGGGATCC**

Codon optimised p47phox

**ATGGGCGACACCTTCATCCGGCACATTGCCCTGCTGGGCTTCGAGAAGAGATTCGTGCCCAGCCAGCACTACGTGTACATGTTTCTCGT
GAAGTGGCAGGACCTGAGCGAGAAGGTGGTGTACCGGCGGTTCACCGAGATCTACGAGTTCCACAAGACCCTGAAAGAGATGTTCCC
CATCGAGGCTGGCGCCATCAACCCCGAGAACCGGATCATCCCTCATCTGCCTGCCCCCAAGTGGTTCGACGGACAGAGAGCCGCCGA
GAACAGACAGGGCACCCTGACCGAGTACTGCAGCACCCTGATGTCCCTGCCCACCAAGATCAGCCGGTGCCCCCATCTGCTGGATTTC
TTCAAAGTGCGGCCCGACGACCTGAAGCTGCCCACCGACAACCAGACCAAGAAGCCCGAGACATACCTGATGCCCAAGGACGGCAA
GAGCACCGCCACCGATATCACCGGCCCCATCATCCTGCAGACCTACCGGGCCATTGCCAACTACGAGAAAACCAGCGGCAGCGAGAT
GGCCCTGAGCACAGGCGACGTGGTGGAGGTGGTGGAAAAGTCCGAGAGCGGCTGGTGGTTCTGCCAGATGAAGGCCAAGAGAGGCT
GGATCCCCGCCAGCTTCCTGGAACCCCTGGACAGCCCTGACGAGACAGAGGACCCCGAGCCTAATTACGCCGGCGAGCCTTACGTGG
CCATCAAGGCCTACACAGCCGTGGAAGGCGACGAGGTGTCACTGCTGGAAGGCGAGGCCGTGGAAGTGATCCACAAGCTGCTGGAC
GGGTGGTGGGTCATCCGGAAGGATGACGTGACCGGCTACTTCCCCAGCATGTACCTGCAGAAAAGCGGCCAGGACGTGTCCCAGGCC
CAGAGACAGATTAAGAGAGGCGCCCCTCCCAGGCGGAGCAGCATCAGAAATGCCCACAGCATCCACCAGCGGAGCCGGAAGAGACT
GAGCCAGGATGCCTACCGGCGGAACAGCGTGCGGTTCCTGCAGCAGAGAAGAAGGCAGGCCAGACCCGGCCCTCAGAGCCCTGGAT
CTCCTCTGGAAGAGGAACGGCAGACCCAGAGAAGCAAGCCCCAGCCTGCTGTGCCCCCTAGACCTAGCGCCGACCTGATCCTGAACC
GGTGCAGCGAGAGCACCAAGAGAAAGCTGGCCTCCGCCGTGTGA**

Truncated codon optimised p47phox

***CCGCCAGCTTCCTGGAACCCCTGGACAGCCCTGACGAGACAGAGGACCCCGAGCCTAATTACGCCGGCGAGCCTTACGTGG
CCATCAAGGCCTACACAGCCGTGGAAGGCGACGAGGTGTCACTGCTGGAAGGCGAGGCCGTGGAAGTGATCCACAAGCTGCTGGAC
GGGTGGTGGGTCATCCGGAAGGATGACGTGACCGGCTACTTCCCCAGCATGTACCTGCAGAAAAGCGGCCAGGACGTGTCCCAGGCC
CAGAGACAGATTAAGAGAGGCGCCCCTCCCAGGCGGAGCAGCATCAGAAATGCCCACAGCATCCACCAGCGGAGCCGGAAGAGACT
GAGCCAGGATGCCTACCGGCGGAACAGCGTGCGGTTCCTGCAGCAGAGAAGAAGGCAGGCCAGACCCGGCCCTCAGAGCCCTGGAT
CTCCTCTGGAAGAGGAACGGCAGACCCAGAGAAGCAAGCCCCAGCCTGCTGTGCCCCCTAGACCTAGCGCCGACCTGATCCTGAACC
GGTGCAGCGAGAGCACCAAGAGAAAGCTGGCCTCCGCCGTGTGA***
