# Supplementary material for: Lentiviral gene therapy rescues p47phox chronic granulomatous disease and the ability to fight Salmonella infection in mice
Source: Gene Ther. 2020 Jun 12;27(9):459–69. doi: 10.1038/s41434-020-0164-6 (PMC7500983; doi:10.1038/s41434-020-0164-6)
Supplement: Supplementary file 3 — Supplementary Figure 2 [file 41434_2020_164_MOESM3_ESM.pdf]

Supplementary Figure 2

A

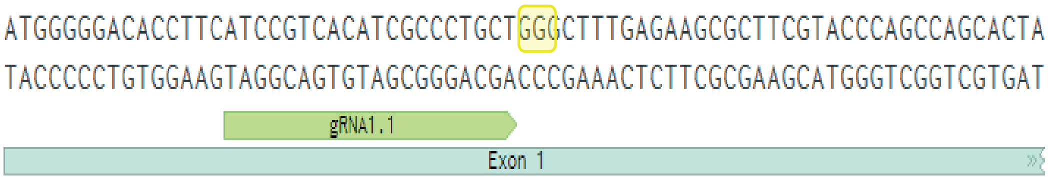

B

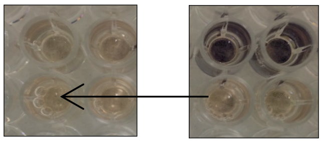

-DMF +DMF

C

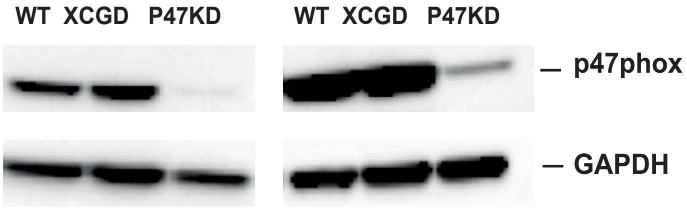

-DMF +DMF

D

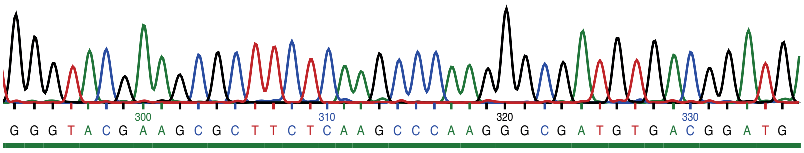


```
GGGTACGAAGCGCTTCTCAA-GCCCA--AGGGCGATGTGACGGATG
|||||||||||||||||||| |||||  ||||||||||||||||||
GGGTACGAAGCGCTTCTCAAAGCCCAGCAGGGCGATGTGACGGATG
```

MUT

```
M  G  D  T  F  I  R  H  I  A  L  G  L  E  K  R  F  V
ATGGGGGACACCTTCATCCGTCACATCGCCCTTGGGCTTGAGAAGCGCTTCGT
```

WT

```
 M  G  D  T  F  I  R  H  I  A  L  L  G  F  E  K  R  F  V
CATGGGGGACACCTTCATCCGTCACATCGCCCTGCTGGGCTTTGAGAAGCGCTTCGT
```
