# Supplementary figures and images for: Lentiviral gene therapy rescues p47phox chronic granulomatous disease and the ability to fight Salmonella infection in mice
Source: Gene Ther. 2020 Jun 12;27(9):459–69. doi: 10.1038/s41434-020-0164-6 (PMC7500983; doi:10.1038/s41434-020-0164-6)

Supplementary Figure 3

A

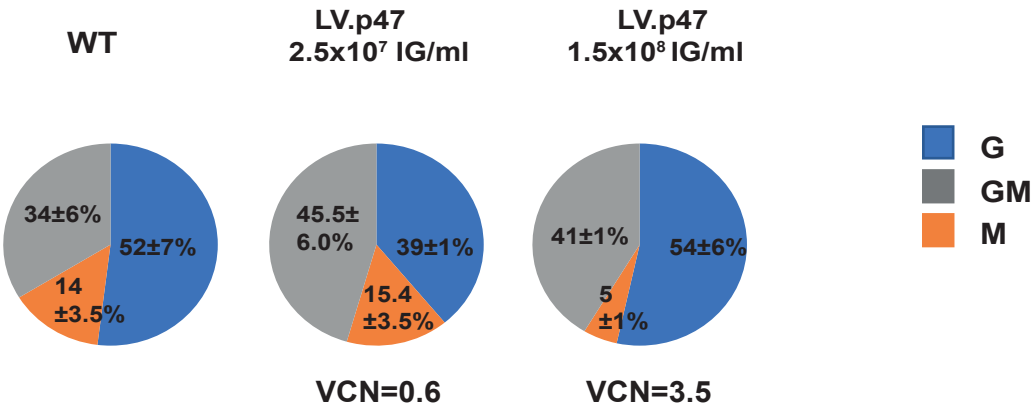

B

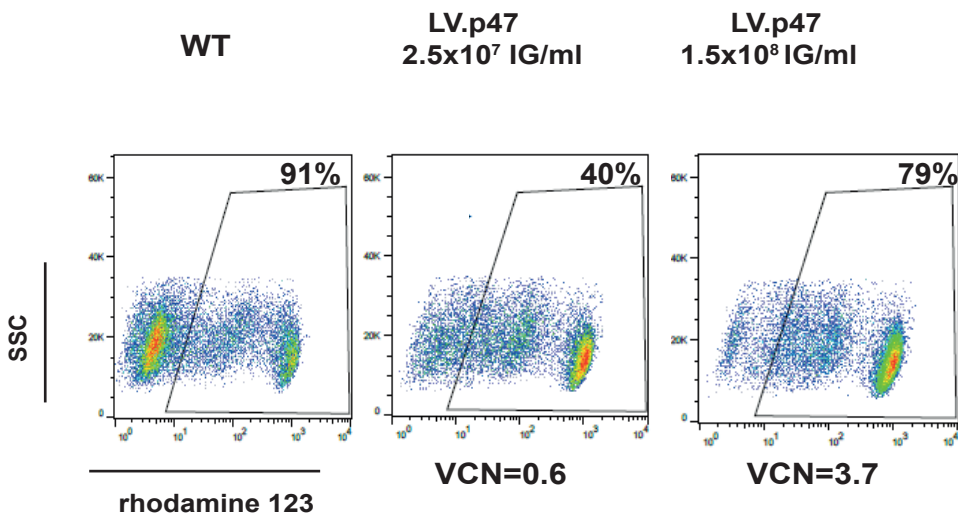

Supplement: Supplementary file 4 — Supplementary Figure 3 [file 41434_2020_164_MOESM4_ESM.pdf]

Supplementary Figure 4

A

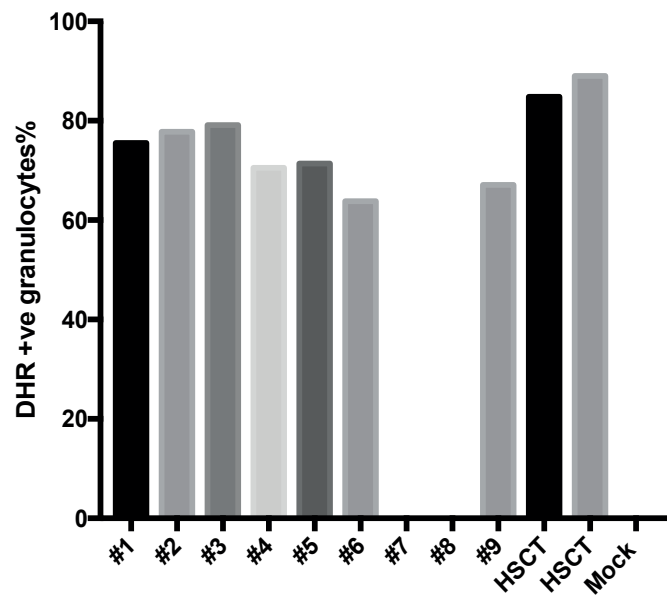

B

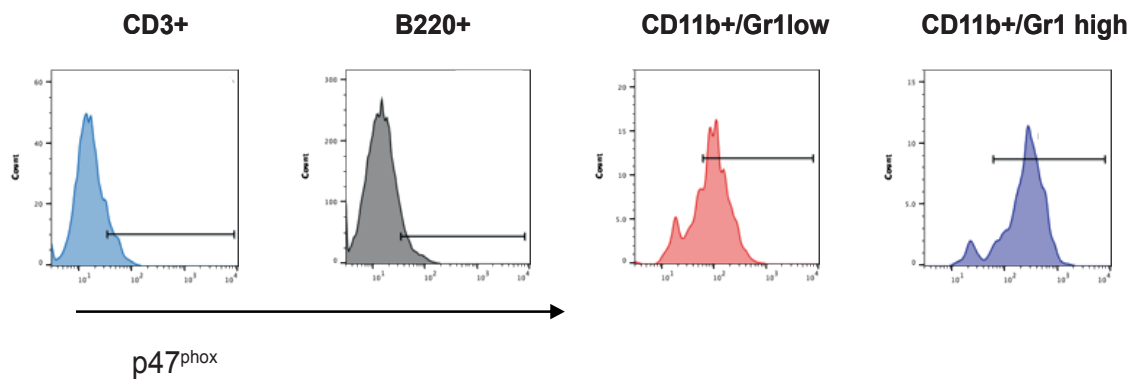

C

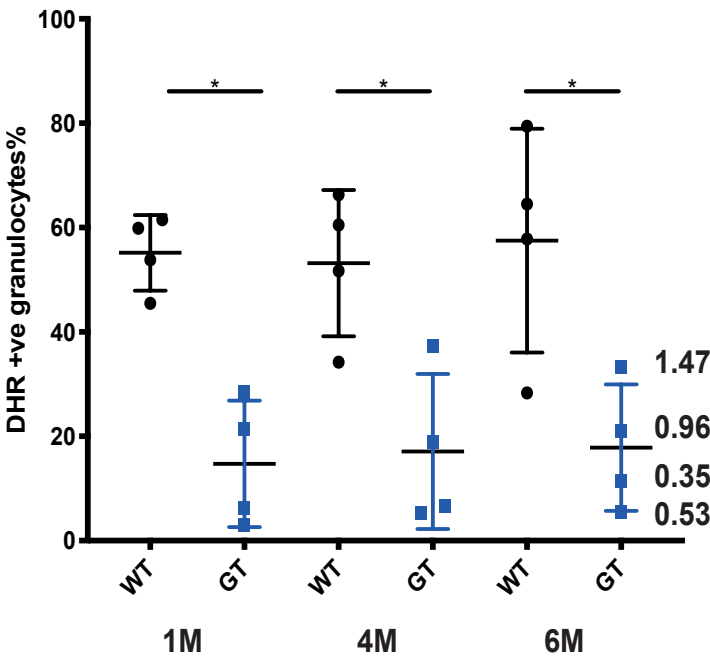

Supplement: Supplementary file 5 — Supplementary Figure 4 [file 41434_2020_164_MOESM5_ESM.pdf]
