# Supplementary material for: Lentiviral gene therapy rescues p47phox chronic granulomatous disease and the ability to fight Salmonella infection in mice
Source: Gene Ther. 2020 Jun 12;27(9):459–69. doi: 10.1038/s41434-020-0164-6 (PMC7500983; doi:10.1038/s41434-020-0164-6)
Supplement: Supplementary file 6 — Supplementary Figure Legends [file 41434_2020_164_MOESM6_ESM.docx]

**Supplementary Information**

**Supplementary Figure Legends**

**Supplementary Figure 1. Transcriptional cassette of the pCCLCHIM-p47^phox^ and the pCCLCHIM-Δ600p47^phox^ vectors**

Shown are the sequences of the Chimeric promoter and those of the full-length codon optimised p47^phox^ and its truncated (Δ600) form (*italics*).

**Supplementary Figure 2. CRISPR/Cas9-generated p47^phox^ defective PLB985 cells (p47KD).** A) Schematics of the binding site of the gRNA targeting the *NCF1* exon 1. The yellow box indicates the PAM sequence. B) Representative Nitroblue tetrazolium test (NBT) on mirror plates from gene-edited clones that underwent myeloid differentiation using Di-methyl-formamide (DMF). Yellow clones are deficient for NADPH oxidase activity. C) p47^phox^ expression detected by immunoblotting in p47KD, wild type (WT) and XCGD PLB985 cells before and after myeloid differentiation. GAPDH was used as a loading control for protein normalization. D) Sanger sequencing showing the homozygous mutations found in the *NCF1* exon 1 of the p47KD clone (in reverse complement) and the corresponding amino acid changes/deletion (L12G, G13L, F14del).

**Supplementary Figure 3. Efficacy of** **lentiviral gene therapy in murine p47^phox-/-^ cells.** A) CFUs counts from WT and virally transduced p47^phox-/-^ lineage negative cells that have been cultured in semisolid medium MethoCult M3534 (StemCell Technologies) supplemented with myeloid cytokines. Shown are vector copy numbers in pooled colonies. B) Representative DHR FACS plots (out of two different experiments). Shown are vector copy numbers in liquid cultures.

**Supplementary Figure 4. Efficacy of lentiviral gene therapy in p47^phox^ null mice**

A) FACS plots showing DHR positive granulocytes in the blood of gene therapy-treated (GT) animals, one month after gene therapy. #9 mouse died during a bleeding procedure 3 months post gene therapy. B) FACS plots showing p47^phox^ expression in T cells (CD3^+^), B cells (B220^+^), monocytes (CD11b^+^/Gr1^low^) and granulocytes (CD11b^+^/Gr1^high^) in a representative peripheral blood sample from a gene therapy-treated mouse out of six. C) Percentage of DHR positive granulocytes found in the granulocytes of peripheral blood of secondary transplanted p47^phox-/-^ mice (GT n=4) and of WT transplanted (WT n=4) at 1, 4, 6 months post transplantation (1M, 4M, 6M). Bone marrow cells from two animals (#1, #2 primary transplant experiment B in table 1) were used for the secondary transplantation experiment. Vector copy number is indicated for each mouse next to the DHR value at 6M.
